# Advances in the Diagnosis of Invasive Pulmonary Mold Infections: Focus on Diagnostic Performance and Cost-Effectiveness of Diagnostic Tests

**DOI:** 10.3390/diagnostics16091384

**Published:** 2026-05-02

**Authors:** Spyridon Papadimatos, Andreas Tziotis, Panos Arvanitis, Audrey Le-Mahajan, Dimitrios Farmakiotis

**Affiliations:** 1 Division of Colon and Rectal Surgery, Department of Surgery, Beth Israel Deaconess Medical Center, 330 Brookline Ave, Boston, MA 02215, USA; spapadim@bidmc.harvard.edu; 2 Department of Obstetrics and Gynecology, Beth Israel Deaconess Medical Center, 330 Brookline Ave, Boston, MA 02215, USA; atziotis@bidmc.harvard.edu; 3 Department of Neurology, Washington University in St. Louis, 1 Barnes Jewish Hospital Plaza, St. Louis, MO 63110, USA; arvanitis@wustl.edu; 4 Division of Infectious Diseases, Beth Israel Deaconess Medical Center, 330 Brookline Ave, Boston, MA 02215, USA; amahaja1@bidmc.harvard.edu

**Keywords:** invasive pulmonary mold infections, aspergillosis, mucormycosis, diagnostic performance, next-generation sequencing, galactomannan

## Abstract

Invasive pulmonary mold infections (IPMIs) are critical complications in immunocompromised patients, contributing significantly to morbidity and mortality. Diagnosing pathogens like *Aspergillus* species (spp.) and the *Mucorales* remains challenging due to non-specific clinical presentations and the limitations of traditional culture methods. This review provides an up-to-date synopsis of IPMI diagnostic tools, focusing on their diagnostic performance, turnaround time (TAT), and cost-effectiveness. We conducted a narrative review of the current literature regarding clinical evaluation, radiographic findings, invasive diagnostics, and non-invasive assays, including next-generation sequencing (NGS) and volatile organic compounds (VOCs). Chest computerized tomography (CT) remains a vital first step, though classic signs like the “halo” or “reverse halo” are neither sensitive nor specific. Traditional diagnostics are limited by low sensitivity and delayed results. While plasma microbial cell-free DNA (mcfDNA) NGS offers rapid TAT (24–48 h) and high specificity, its suboptimal sensitivity for *Aspergillus* spp. (<50%) and high cost remain significant barriers. Investigational VOC “breath tests” show promising sensitivity (77–96%) but lack standardization. Future research must prioritize the standardization of non-invasive microbiologic testing modalities, particularly those with rapid TAT such as bedside “breath tests” and high-throughput mcfDNA NGS. Development of clinical algorithms that balance cost-effectiveness with timely pathogen diagnosis based on the patient’s degree of immunosuppression is essential to improve survival in high-risk populations.

## 1. Introduction

Despite advances in antifungal therapy, Invasive pulmonary mold infections (IPMIs) remain among the most severe infectious complications in hematopoietic cell transplant (HCT), and organ transplant (OT) recipients, patients with hematologic malignancies (HM), and patients receiving immunosuppressive agents, contributing substantially to morbidity and mortality [1,2]. *Aspergillus* spp. and the *Mucorales* are the leading culprit pathogens, while *Fusarium* spp. and *Scedosporium* spp. are less common but commonly associated with antifungal resistance and poorer outcomes [3,4,5]. Mortality rates remain high across all IPMIs, reaching up to 90% in certain clinical scenarios [3].

**Figure 1:**
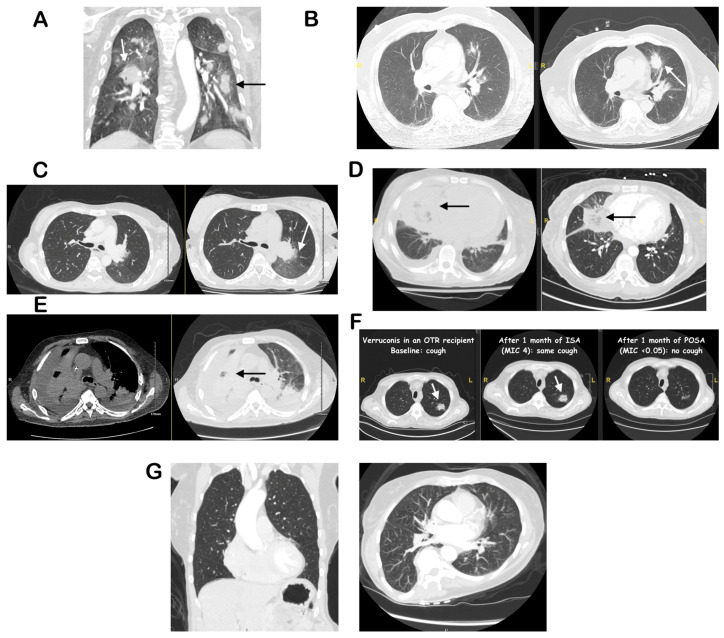
Arrows indicate characteristic abnormal findings. (**A**): Multifocal consolidations in the setting of high-dose steroids, serum GM > assay cut-off (OD 8), autopsy showed angioinvasive septate narrow-angled hyphae, consistent with IPA. (**B**): Enlarging nodule with “halo sign” in a neutropenic patient with acute myeloid leukemia, positive serum bDG, BAL PCR and plasma mcfDNA positive for *Aspergillus fumigatus*. (**C**): Nodule with “halo sign” in a neutropenic patient with aplastic anemia, positive BAL Galactomannan and plasma mcfDNA positive for *Aspergillus flavus*/*oryzae*. (**D**): “Reverse Halo” sign with rapid progression off treatment, negative BAL and tissue biopsies. Plasma mcfDNA-NGS was positive for *Rhizopus delemar* and autopsy showed angioinvasive aseptate broad-angled hyphae consistent with mucormycosis. (**E**): Large consolidation with BAL growth of a *Syncephalastrum* species (not further speciated). (**F**): Multiple pulmonary nodules with BAL growth of *Verruconis galopava* (“black” mold), and response to different anti-mold agents (OTR: Organ transplant recipient, POSA: Posaconazole, ISA: Isavuconazole), correlating with its minimal inhibitory concentrations (MIC). (**G**): Normal chest CT.

Defects in neutrophil function and, less so, cell-mediated immunity are the major risk factors for IPMIs [2,4,6]. In immunocompromised patients, the absence of typical signs and symptoms such as fever, cough, and dyspnea can delay diagnosis [2,7]. Certain radiographic findings, including the “halo” and “air-crescent” signs suggestive of invasive pulmonary aspergillosis (IPA) and the “reverse halo” sign or pleural effusions suggestive of mucormycosis, may assist in diagnosis [6,8,9] (Figure 1). However, these findings are not always present (thus, not sensitive) and can overlap with other IPMIs and opportunistic pulmonary syndromes (thus, not specific). Traditional microbiologic diagnostic methods, including fungal stains, cytology, and bronchoalveolar lavage (BAL) or tissue culture, although widely available, are limited by low sensitivity, need for invasive sampling, dependence on organism burden, and delayed turnaround times (TATs) [5,7].

Non-culture-based fungal diagnostics, like serum galactomannan (GM, positive if >0.5 [optical density, OD]) for IPA, β-D-glucan (BDG) for invasive candidiasis and other fungal infections, and mold-specific polymerase chain reaction (PCR), have improved early detection of certain IPMIs, but need to be interpreted carefully due to variable specificity and potential cross-reactivity [10,11,12]. Next-generation sequencing (NGS) of microbial cell-free DNA (mcfDNA)-based assays in plasma and BAL allow for rapid, non-invasive and culture-independent identification of invasive molds. However, clinical validation remains limited, and cost and availability pose important barriers to widespread use [1,13,14,15].

In this narrative review, we present an up-to-date synopsis of diagnostic tools for IPMIs in immunocompromised hosts, focused on diagnostic performance, TAT, and cost considerations for conventional and novel tests.

## 2. Clinical Evaluation

Diagnosing IPMIs is almost impossible by clinical exam alone. Identifying host risk factors is the first, and often the most important step. Prolonged neutropenia has been most strongly associated as an independent risk factor. Several other “immunomodulating” agents, such as Bruton’s tyrosine kinase and JAK inhibitors have emerged as predisposing factors to IPMIs through poorly understood off-target effects [9,15]. Patients with prolonged intensive care unit (ICU) stay are also at risk for IPMIs, especially IPA, even without any other classic risk factors. Early symptoms and signs of IPMIs are usually mild and nearly always non-specific, overlapping with other infections and underlying HM, such as cough (usually dry), fever and malaise [1,2,4,7,13]. Chest pain can occur with tissue infarction. The physical exam is often unrevealing, although sometimes can reveal skin [16] or oral (hard palate eschar from invasive sinusitis in the setting of sino-orbital or rhinocerebral mucormycosis) [17] lesions, suggesting an invasive mold infection. It should be noted that all details of medical history are important because in many patients underlying medical problems can confound the differential diagnosis and management decisions. For example, frequent thrombocytopenia makes invasive testing risky or not feasible.

## 3. Radiographic Findings

Computerized tomography (CT) of the chest is far more sensitive than chest X-ray (CXR), gives results fast (within hours), and, although costly (up to >$4000 depending on the type and protocol used), is widely available, even outside referral hospitals, and indicated as the first step in all patients with suspected IPMIs, because it can identify pulmonary lesions as the cause of the patient symptoms and can guide the areas to undergo BAL or even biopsy.

The term “halo sign” describes a pulmonary nodule surrounded by ground glass attenuation, whereas the “vessel occlusion sign” refers to the interruption of a pulmonary artery branch within a lesion. Both signs reflect angioinvasion with surrounding hemorrhage, and can be observed in IPMIs, especially IPA (Figure 1). Cavities, and especially the “air crescent” sign are also considered suggestive of IPA but can be present in other fungal and bacterial infections. The reverse halo sign, a rounded area of “ground-glass” opacities surrounded by a ring of consolidation, and the “bird’s sign”, a reverse halo with intersecting internal strands, are caused by central necrosis and are considered diagnostic of angioinvasive mucormycosis, especially in neutropenic patients (Figure 1) [8].

In one study, multiple nodules and pleural effusion(s) differentiated IPA from pulmonary mucormycosis (PM) [18]. In another, more recent report, large consolidative lesions and absence of airway invasion, as well as the reverse halo sign differentiated PM from IPA [19] (Figure 1). Nonetheless, in patients with uncontrolled diabetes, PM can also present with endobronchial, exophytic lesions [17]. Notably, chest CT with angiography can reliably rule out IPMIs by absence of vessel infarction [20,21,22].

It should be again noted that such signs are non-specific, and may also occur in other lung diseases such as bacterial infections, organizing pneumonia or tuberculosis. Furthermore, in non-neutropenic immunocompromised patients, the classic signs seen on chest CT are frequently absent, and diagnostic imaging can be more challenging; in such cases, chest CT may be supplemented by other imaging techniques, such as CT angiogram or positron emission tomography (PET) CT, when clinically indicated [23]. In ICU patients, diffuse infiltrates or acute respiratory distress syndrome (ARDS)-like changes can mask the underlying diagnosis, making CT interpretation more challenging [9]. PET CT is increasingly used for staging and monitoring the response of IPMIs to treatment, but its use for infection surveillance is not routinely recommended.

## 4. Invasive Diagnostics

The “gold standard” for diagnosis of IPMIs requires invasive bronchoscopy with tissue biopsy, though this cannot be performed safely in patients who are at high risk for respiratory decompensation or major bleeding [1,2]. If unable to obtain tissue specimens, the next highest quality microbiologic evidence for IPMIs would be acquired via bronchoscopy with bronchoalveolar lavage (BAL). Importantly, traditional culture positivity in addition to diagnosis, allows for susceptibility testing (Figure 1F). A “probable” diagnosis of IPMIs can be made if BAL fungal tests are positive in the right host with the right radiographic findings [23]. Use of respiratory specimens other than BAL, like sputum, or endotracheal aspirate, may be pursued, but obtaining adequate quantity and quality specimens are essential, given high risk of detecting colonization and overall lower diagnostic yield [9,23]. Molecular methods (PCR or NGS) from respiratory samples usually have better sensitivity than cultures and faster TAT [5,10,14]; however, lack of PCR platform standardization still leads to variable accuracy across laboratories. It is also difficult to know if a positive result represents true infection or non-pathogenic airway colonization, especially for *Aspergillus* spp. [9]. A multiplex PCR panel for *Aspergillus* spp., the *Mucorales*, *Nocardia* spp. and *Pneumocystis jirovecii* is commercially available in the US (Viracor-Eurofins, Table 1). One study showed poor sensitivity (31%) but high specificity (97%) for aspergillosis [24]. In the same study, specificity for the *Mucorales* and *Nocardia* spp. was 100% (no confirmed false positives), but neither were identified by the standard of care, despite compatible clinical syndromes; therefore, the sensitivity could not be calculated. Another study [9] looked at panfungal and *Mucorales* PCR assays in several biological samples, including tissue from biopsy, and BAL was 100% sensitive and 91% specific. Further studies are needed to determine the diagnostic yield and negative predictive value of such assays.

Assays based on mcfDNA-NGS can detect a wide range of organisms directly from plasma or BAL [1,13]. These tests are very promising, but expensive, not widely available, and can yield false positives from contamination, colonization, or transient DNAemia [13,14]. The utility of BAL NGS is, therefore, still largely under development. The greatest advantage of NGS is rapid TAT (Table 1). Traditional methods (such as culture, histopathology, and antigen detection) still play a critical role, but are slow and often lack sensitivity.

Serum GM, in the right clinical setting, has excellent specificity and sensitivity for invasive aspergillosis. Likewise, detection of GM in BAL is often indicative of an IPMI, especially from *Aspergillus* spp., but test specificity is dependent on the host (with highest specificity in HM patients). Furthermore, GM can be false positive, especially in the BAL, even with very high values, from other reasons (cross-reactivity with other mannans) including heavy *Candida* colonization [40] or food aspiration [41]. It should be noted that, unlike BAL GM, there is no role in testing BDG levels in the BAL, a test that is largely non-specific for fungal infections and also has very poor reproducibility [42].

Broad-range PCR for bacteria, viruses and fungi in BAL or tissue has been better studied, and is most helpful when stains are positive, but growth is absent (for example, in the setting of antimicrobial treatment [43]). It takes longer than NGS (5–10 vs. 2–3 business days, Table 1) but is less costly, and depending on the libraries built for NGS, can be potentially more sensitive given targeted DNA primers. For tissue samples with visualized hyphae but without corresponding microbiological studies or culture growth, another option for mold identification is immunohistochemistry with mold-specific antibodies at the Centers for Disease Control (CDC) [44].

Cytology testing is another important yet often overlooked component of BAL diagnostics. In addition to helping diagnose malignancy, which is sometimes on the differential (especially with lymphangitic spread that can mimic atypical pneumonia or cavitated tumors), it can also provide a diagnosis of mold infections, since the Gomori Methenamine Silver stain used in histopathology is more sensitive than the Calcofluor stain in the microbiology laboratory [7]. In addition, cytology can supplement direct fluorescent antibody testing for *Pneumocystis*, identify viral cytopathic changes (HSV, CMV) or highlight larvae in *Strongyloides* hyperinfection, which are often important considerations on the differential diagnosis for atypical pulmonary infection in immunocompromised hosts [7,45,46].

## 5. Non-Invasive Diagnostics

Culture provides a definitive diagnosis and allows for antifungal susceptibility testing with potential resistance profiles [9,23]. However, conventional non-invasive sampling for organism growth is limited to blood cultures, which rarely yield mold. The majority of molds do not grow from blood, as most filamentous fungi do not circulate in a viable form nor can they survive in standard broth media [47]. Exceptions are *Fusarium* spp. and *Scedosporium*/*Lomentospora* spp., which are capable of producing conidia in the bloodstream [9,23]. Detection of these molds in blood cultures generally reflects disseminated disease [48].

With regards to non-culture diagnostics, serum GM and BDG are helpful but largely imperfect. GM, a polysaccharide released from the cell wall of *Aspergillus* spp. during hyphal growth, can be used as a genus-specific biomarker for IPA. The assay has been validated for use in serum and BAL, although detection in other tissues (e.g., plasma, CSF) may also support the diagnosis of IPA when compatible with clinical and radiologic findings [9,23]. Diagnostic thresholds consistent with probable infection include a GM index of ≥1.0 in serum, plasma, BAL fluid, cerebrospinal fluid (CSF), or a serum or plasma index ≥ 0.7 combined with a BAL index ≥ 0.8 [23]. The test’s sensitivity varies widely, ranging from 20% to 90%, depending on host factors, fungal burden, and specimen type. Exposure to mold active antifungal agents substantially reduces assay sensitivity [9,23].

GM is quite specific for *Aspergillus*, but false positives can occur with certain antibiotics or foods, and results are less reliable in non-neutropenic patients [11]. It should be noted that with recent modifications in antibiotic manufacturing, especially piperacillin/tazobactam, the risk for false positive cross-reactivity from antibiotics is considered minimal [49]. In the largest meta-analysis of GM diagnostic performance today, its sensitivity and specificity among patients with hematologic malignancies were 92 and 90%, respectively. Sensitivity increased to 99% with either positive GM or non-invasive (serum) PCR for *Aspergillus*, and specificity was 95% and 98% with two positive GM or positive GM and PCR, respectively, indicating potential utility for repeat or combined blood testing [50].

The BDG assay is a panfungal antigen test that detects a cell wall polysaccharide present in most pathogenic fungi (including *Pneumocystis*), with the exceptions of *Cryptococcus*, *Blastomyces*, and the *Mucorales* [9,11,12,23]. As such, it is not specific for diagnosing IPMIs, nor for any specific fungal pathogen. As another caveat, BDG is almost always (falsely) positive after IVIg administration, and can also be falsely elevated in hemodialysis or bacterial sepsis [12].

*Aspergillus* spp. PCR assays, although not yet as standardized or widely used as GM, constitute a robust diagnostic tool for both screening and confirmation of IPA as they are both sensitive and specific [9]. Current evidence supports their use on serum, plasma, whole blood, and BAL fluid, with the strongest data derived from studies in patients with hematologic malignancies and those undergoing HCT [9,23]. Diagnostic criteria consistent with probable infection include two or more consecutive positive PCR tests from plasma, serum, or whole blood, two or more positive replicate tests from BAL fluid, or at least one positive blood-based PCR in combination with one positive BAL PCR [23]. As previously mentioned, the combined use of PCR and GM can substantially increase the diagnostic yield of non-invasive testing when IPA is suspected. Importantly, PCR assays have the unique ability to detect *Aspergillus* at both the genus and species levels. Furthermore, some platforms may also offer the potential to identify mutations associated with triazole resistance making them potentially valuable in settings where azole resistance is prevalent [23].

Beyond aspergillosis, blood PCR can be used to diagnose mucormycosis. In a prospective study including several susceptible hosts (mostly with HM, but also OT and diabetes) with suspected mucormycosis, the *Mucorales* quantitative PCR assay, demonstrated 85.2% sensitivity and 89.9% specificity for the diagnosis of proven or probable mucormycosis [9,35].

One promising and rapidly evolving methodology to diagnose IMI and other elusive infectious syndromes is non-invasive NGS in plasma. Two tests are commercially available in the US: The NexGen Assay for the detection of fungi/mycobacteria/*Nocardia* spp. (Viracor-Eurofins), and the “Karius” microbial cell-free DNA metagenomic NGS assay (Karius Inc., Redwood City, CA, USA) that can detect any of >1000 DNA pathogens in blood (Karius Spectrum—KS) or BAL (Karius Focus—KF), including almost all medically important molds. Whereas clinical data on NexGen are limited to an ongoing open-label, investigator-initiated study, several studies recently summarized have highlighted the utility of KS in diagnosing IMI, but also the assay’s notable shortcomings [51].

The specificity for KS in IPMI is excellent (nearly 100% across studies) [51,52,53]; however, its sensitivity for the most common IPMI, IPA has been consistently reported <50% [51,52,53]. In the aforementioned study, the denominators of proven/probable IPA (host + clinical criteria but also +GM) could include cases with false +GM; however, mcfDNA NGS in plasma likely has suboptimal sensitivity for the detection of *Aspergillus* spp., and potentially other less angioinvasive than the *Mucorales*. The reasons for these observations are still unclear, but likely related to the structure of mold DNA, variable angioinvasion and burden of disease, and the need for computational algorithms to remove human (=other eukaryotic) DNA and exclude lab contamination of the specimen with *Aspergillus* spp.

These limitations do not apply to the same extent to the more angioinvasive *Mucorales*, and the sensitivity of KS is higher for mucormycoses, as high as 100% in a recent case series [53], although sensitivity of KS for diagnosis of early or more localized mucormycoses may be lower in real-life. Notably, mcfDNA NGS testing in BAL is expected to have higher sensitivity for pulmonary IMI [52], pending publication of additional high-quality, peer-reviewed data.

The main advantages of mcfDNA NGS, especially in plasma as a non-invasive diagnostic modality are rapid TAT (2–3 business days), high specificity, and persistent detection in the setting of antimicrobial use. An important limitation is the cost.

Several novel technologies are under investigation for the non-invasive diagnosis of fungal pneumonia. One of the most promising is gas chromatography mass spectrometry (GC MS)-analysis of volatile organic compounds (VOCs) produced by the metabolic activity of infecting fungi in exhaled breath (“breath test”), allowing for rapid, non-invasive detection of respiratory fungal and bacterial pathogens [1,9]. In observational studies, GC MS analysis of exhaled metabolites demonstrated sensitivity ranging from 77% to 96% and specificity of 78% to 97% for IPA, particularly in transplant recipients and other immunocompromised hosts [38,54]. The methodology is under development for the detection of other molds, too [1,55,56,57]. As of now, the technology is investigational and requires specialized laboratory equipment, but the ultimate goal is implementation of a portable device that can be used at the bedside. For the time being, its specific diagnostic yield, TAT, and cost-effectiveness remain unknown.

## 6. Conclusions and Future Directions

Major diagnostic advances have been made in the last few years in the care of immunocompromised patients at risk for IPMIs, focusing on two main areas: 1. Advanced, faster diagnostics with higher sensitivity than traditional cultures, especially fungal biomarkers and molecular testing; 2. Implementation of sensitive imaging, mainly high-resolution CT, with or without angiogram protocols to detect angioinvasion, and PET scan. Nevertheless, IPMIs remain an area of major concern in infectious diseases, mainly associated with their high mortality rates, and challenges in timely and accurate diagnosis. Unfortunately, IPMIs are still often surprisingly diagnosed at autopsy [40,58,59], highlighting the need to better understand host risk factors and enhance our capacity for early, accurate, non-invasive diagnostic modalities with short TAT.

Therefore, future research is needed to focus on (a) the development of such novel diagnostic methods, including “breath tests”; (b) further characterization of the specificity, sensitivity, predictive values and cost-effectiveness of costly, high throughput NGS assays with rapid TAT; (c) development and validation of specific diagnostic algorithms that factor into clinical decision-making the cost of different (serial) tests and the ideal time-to-pathogen diagnosis, across different hosts and urgency to diagnose (e.g., plan for imminent added immunosuppression as in allo-HCT candidates) clinical scenarios.

## Figures and Tables

**Table 1:** Diagnostic modalities for invasive pulmonary mold infections in immunocompromised patients.

Category	Test/Modality	Specimen	Typical TAT	Sensitivity (%)	Specificity (%)	Approximate Cost (USD, 2024–2025) *	
Microscopy & Histopathology	Direct stains (GMS, PAS, calcofluor)	BAL, sputum, tissue	Minutes–hours	<50	High when tissue invasion present	~$10–30 per specimen	[25,26,27]
Microscopy & Histopathology	Histopathology (FFPE or fresh tissue)	Biopsy	Hours–1 day	Variable (sampling dependent)	High for tissue invasion; demonstrates angioinvasion	~$100–200 per biopsy (pathology technical fee)	[25,26,27]
Culture-Based Diagnostics	Fungal culture	BAL, sputum, tissue, sterile fluids	1–7 days (up to 21 days for slow growers)	~50 overall; <30 in respiratory IPA	~100 (species-level identification)	~$30–60 per culture set	[25,27,28]
Culture-Based Diagnostics	Blood culture for molds	Blood	1–7 days	Low overall; higher for *Fusarium*/*Scedosporium* spp.	High; usually indicates disseminated disease	~$60–120 per set (aerobic/anaerobic bottles)	[25,27,28]
Culture-Based Diagnostics	MALDI-TOF MS (from culture)	Culture isolate	Minutes once colony available	Same as culture yield	~100 (species-level identification)	Incremental ~$5–15 per isolate (reagent cost)	[25,29]
Targeted Molecular/Tissue-Based Tests	PCR + sequencing (species-level)	FFPE or fresh tissue	1–3 days	High when fungal elements seen on histology	High; useful for non-distinct morphology or culture-negative disease	~$250–400 per assay	[25,30]
Non-Culture Biomarkers	Serum galactomannan (GM)	Serum, plasma	1–3 days	20–90 (host- and specimen-dependent)	~80–90	~$100–200 per test	[25,27,31]
Non-Culture Biomarkers	BAL galactomannan	BAL fluid	1–3 days	Up to ~90	~90	~$120–220 per test	[25,32]
Non–Culture Biomarkers	(1→3)-β-D-glucan (BDG)	Serum	Same day–1 day	~60–80 (highest in hematologic/HSCT)	~70–90; reduced in ICU due to false positives	~$400–450 per test	[25,33]
Fungal PCR (Pathogen-Specific)	*Aspergillus* PCR	Serum, plasma, whole blood, BAL	1–2 days	70–100	85–95	~$200–350 per assay	[25,34]
Fungal PCR (Pathogen-Specific)	*Mucorales* PCR	Serum, BAL, tissue	1–2 days	~85	~89–90	~$200–350 per assay	[35]
Broad Molecular & Advanced Diagnostics	Broad-range fungal PCR	Tissue, sterile fluids	1–3 days	50–70	High; adjunct when microscopy positive but cultures/PCR negative	~$250–450 per assay	[25,30]
Broad Molecular & Advanced Diagnostics	Plasma microbial cell-free DNA sequencing/mNGS	Plasma (±BAL, tissue)	24–48 h	40–70	80–90	~$1800–2500 per test	[36,37]
Emerging Non-invasive Technologies	Exhaled VOCs by GC–MS	Exhaled breath	Minutes–hours	77–96 (IPA and CPA)	78–97	Research only; estimated reagent cost ~$200–400 per run	[38]
Imaging	High-resolution CT (HRCT) chest	Imaging	Immediate	High for typical angioinvasive patterns	Limited; findings often non-specific in non-neutropenic hosts	~$130–200 (Medicare technical payment) to >$500 list price	[27,39]

* Approximate direct test costs in US dollars based on 2024–2025 cash prices, manufacturer information, and fee-schedule estimates where available; values illustrate relative order of magnitude rather than provide exact charges. Actual costs vary between institutions, health systems, countries, and payers and may differ substantially from the amounts billed to or paid by patients and insurers.

## Data Availability

No new data were created or analyzed in this study.

## Abbreviations

The following abbreviations are used in this manuscript:

IPMI	Invasive pulmonary mold infection
HCT	Hematopoietic cell transplant
OT	Organ transplant
HM	Hematologic malignancies
IPA	Invasive pulmonary aspergillosis
BAL	Bronchoalveolar lavage
TAT	Turnaround time
GM	Galactomannan
BDG	Beta-D-glucan
PCR	Polymerase chain reaction
NGS	Next-generation sequencing
mcfDNA	Microbial Cell-free DNA
ICU	Intensive care unit
CT	Computed tomography
CXR	Chest X-ray
PET	Positron emission tomography
GGO	Ground-glass opacity
GMS	Gomori methenamine silver
HSV	Herpes simplex virus
CMV	Cytomegalovirus
CDC	Centers for Disease Control and Prevention
VOCs	Volatile organic compounds
GC-MS	Gas chromatography–mass spectrometry
IVIg	Intravenous immunoglobulin
ARDS	Acute respiratory distress syndrome
PM	Pulmonary mucormycosis
